# Hyperammonemia of unknown cause in a young postpartum woman: a case report

**DOI:** 10.1186/s13256-022-03304-y

**Published:** 2022-03-07

**Authors:** Sadaf Hanif, Sher Muhammad Sethi

**Affiliations:** 1 Internal Medicine and Critical Care, Aga Khan University Hospital, Karachi, Pakistan; 2 Resident III, Internal Medicine, Aga Khan University Hospital, Karachi, Pakistan

**Keywords:** Ammonia, Hyperammonemia, Urea cycle disorder, Ornithine carbamoyltransferase

## Abstract

**Background:**

Hyperammonemia is a medical condition described as increased or elevated serum ammonia levels. High serum levels of ammonia can cause neurotoxicity. Sudden onset severe hyperammonemia may cause severe encephalopathy with brain damage. It can result in cerebral edema, emesis, seizures, hypotonia, and death. We report a young postpartum woman who had a sudden rise in serum ammonia levels after vaginal delivery.

**Case presentation:**

A 24-year-old, married, postpartum Pakistani woman was admitted to the intensive care unit through the emergency department, with complaints of fever, severe abdominal pain with distension, and altered levels of consciousness. The patient had a medical history of spontaneous vaginal delivery 2 weeks before this hospital admission, after which she gradually developed the above symptoms. However, the patient’s past medical history was unremarkable with no hepatic disease, but her investigations revealed a progressive rise in serum ammonia levels. In the intensive care unit, she developed generalized tonic–clonic seizures. This was followed by a coma, tonsillar herniation, and death.

**Conclusion:**

Postpartum hyperammonemia is a rare entity. It is a critical illness and must be evaluated for underlying metabolic disorders. Early diagnosis and treatment may result in better outcomes and reduced mortality among postpartum women with hyperammonemia.

## Introduction

Ammonia is a metabolite produced in the human body as a result of protein and amino acid breakdown. Ammonia also aids in the synthesis of amino acids and maintains acid–base balance. However, high levels of ammonia can cause severe toxicity [1, 2].

Hyperammonemia refers to an elevated or increased level of serum ammonia in the body. High levels in the serum may result in neurotoxicity [2]. Sudden-onset severe hyperammonemia may cause severe encephalopathy with brain damage. Furthermore, cerebral edema, emesis, seizures, hypotonia, and death can occur [2, 3]. However, a chronic, mild rise in ammonia levels can cause neuropsychiatric problems, such as delirium and behavioral changes. Survivors of severe neonatal hyperammonemia suffer structural brain damage [3].

Hyperammonemia is present among adult patients with complicated liver disease. The metabolic disorders causing hyperammonemia include deficiencies of urea cycle enzymes, citrin, and pyruvate carboxylase [4, 5]. In critical care units, most patients with hyperammonemia present with an acute rise in ammonia, which requires immediate management to prevent brain damage and death. The other causes of hyperammonemia include ornithine carbamoyltransferase deficiency, hematologic malignancy, and the side effects of valproic acid and 5-fluorouracil (5-FU) [5].

Here we report a case of young postpartum women who had a sudden rise in ammonia levels after vaginal delivery.

## Case presentation

A 24-year-old postpartum Pakistani woman was admitted to the intensive care unit (ICU) of a well-equipped private tertiary care hospital through the emergency department. The presenting complaints were fever, severe abdominal pain with distension about 2 weeks after delivery, and altered levels of consciousness (ALOC) presenting 2 days before admission. She had a high-grade fever, undocumented, without chills and rigors, relieved with medications, and associated with severe generalized abdominal pain. Abdominal pain was sharp, constant, had no aggravating or relieving factors, and was associated with bloating and distension. Over the past 2 days she developed altered mentation in which she was talking inappropriately, not recognizing close family members, and getting drowsier. She was primigravida and had a spontaneous vaginal delivery 2 weeks before this hospital admission, after which she gradually developed the above symptoms. However, she did not have a significant medical history. Family history was also unremarkable. On physical examination, she was pale, tachycardiac (heart rate was 120 bpm), and tachypneic (respiratory rate was 32 breaths/minute). She had a Glasgow Coma scale score of 8/15, so was electively intubated. Her motor examination was unremarkable, while the rest of the neurological exam cannot be accessed due to low mentation. Her cardiovascular and respiratory examinations were insignificant. In the abdomen, examination revealed a soft, distended abdomen with hepatomegaly and positive shifting dullness. There was no tenderness in the abdomen. An investigation was then conducted to determine the cause of her symptoms. Her initial laboratory investigations are presented in Table 1. Based on her high polymorphic leukocytosis on cerebrospinal fluid (CSF) analysis, it was suspected that she had acute bacterial meningitis. Hence, empirical antibiotics in meningitic doses were given. Despite treatment, her central nervous system examination showed no improvement. She remained comatose throughout her ICU stay.

**Table 1:** Laboratory investigation of the patient

Complete blood count	Hemoglobin: 10 mg/dL Hematocrit: 32% Total leukocyte count: 10,000/mL Platelets count: 51,000
Liver function tests	Total bilirubin : 1.7 mg/dL Alanine transaminase: 15 IU/L Aspartate aminotransferase: 38 U/L Gamma-glutamyl transferase: 16 IU/L Alkaline phosphatase: 35 IU/L
Enzymes	Trop-I : Negative Procalcitonin : 8 ng/mL Creatinine phosphokinase: 391 IU/L
Screening for infections	Malaria parasite microscopy: Negative Dengue ELISA : Negative HIV ELISA: Negative Hepatitis B antibody: Nonreactive Hepatitis C antibody: Nonreactive
CFS examination	Protein levels: 25 mg/dL Glucose levels: 72 mg/dL Chloride levels: 136 mg/100 mL Leukocyte count 19 cells/µL (neutrophil 80% and lymphocyte 20%)

**Fig. 1:**
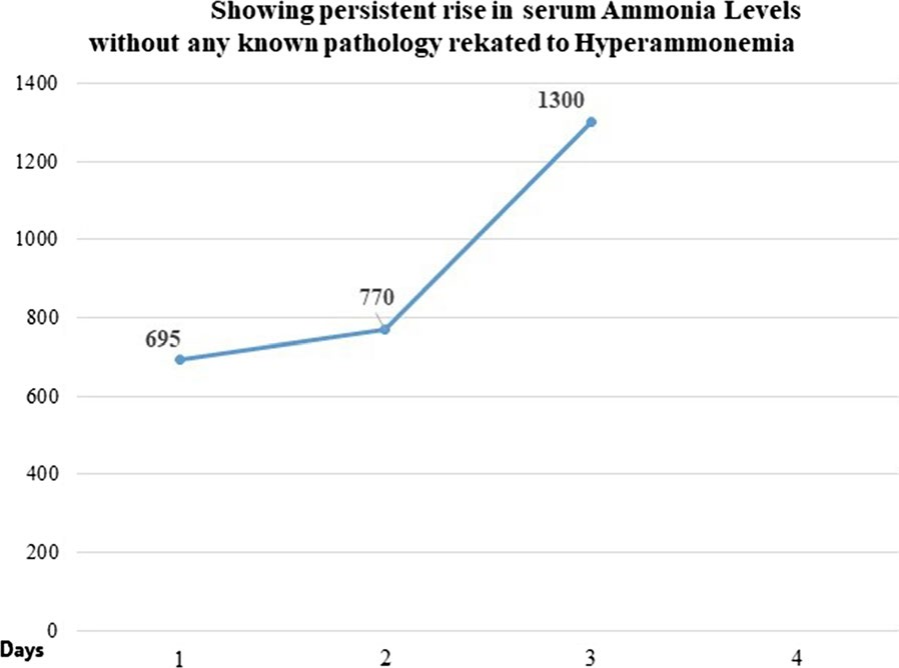
Persistent rise in serum ammonia levels

Moreover, she developed generalized tonic–clonic seizures. Levetiracetam was initiated to control seizures. Her blood biochemistry showed a persistent rise in serum ammonia levels. Three ammonia level assessments showed a gradual increase from 695 µ/dL, 770 µ/dL, to 1300 µ/dL in 3 days (Fig. 1). Ultrasound of the liver showed normal liver size, shape, and echo texture, with no evidence of chronic liver disease.

**Fig. 2:**
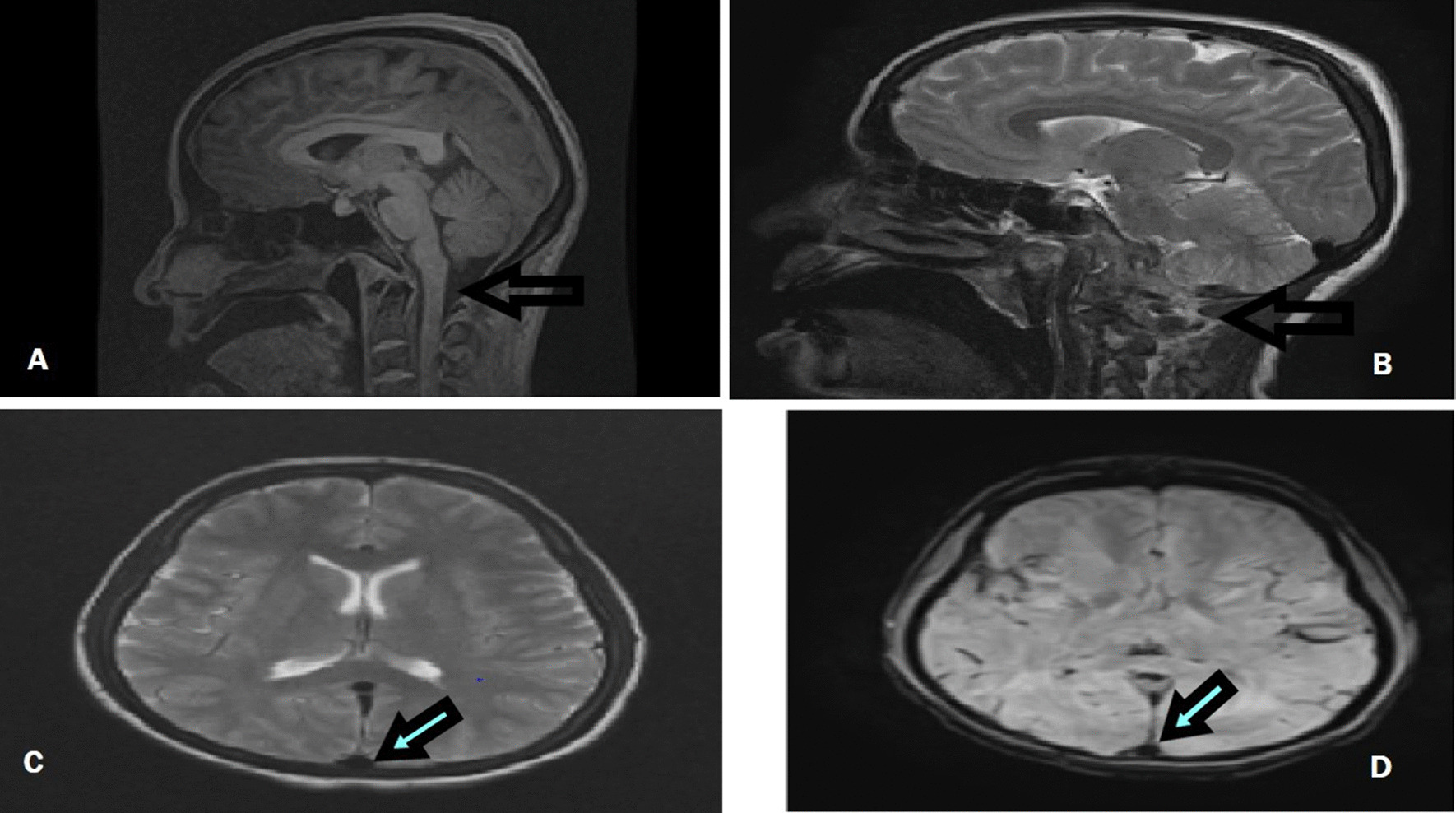
MRI brain showing tonsillar herniation via black arrows on sagital plane (**A**, **B**) and compression of ventricle with slight deviation of cereberum to left is shown via arrows on axial plane (**C**, **D**)

She was kept on a high protein diet to prevent hyperammonemia. A medication to neutralize ammonia was not offered because of nonavailability. She purged with 2–3 stools per day. Lactulose and l-carnitine were given. The nephrology team was taken on board and she had a session of hemodialysis. On the third day of admission, her pupil got fixed. A magnetic resonance imaging of the brain was then done that showed tonsillar herniation (Fig. 2).

Despite conservative management and all efforts to control hyperammonemia, she suffered permanent brain damage. Postpartum examination was not performed. We did not perform any tissue biopsy. Genetic testing is not available at our institution. The patient’s family was informed about the guarded prognosis and they decided to withdraw support. The patient succumbed to death after being removed from the ventilator on 4th day of the hospital stay.

## Discussion

We report a case of a young woman with rapidly progressive hyperammonemia of unknown origin.

Her initial diagnostic workup failed to reveal any relevant etiology including chronic liver disease. Moreover, she was treated for suspected bacterial meningitis but she never responded to the treatment and remained comatose. Furthermore, a rapid rise in serum ammonia level led to further brain damage, which resulted in tonsillar herniation and death. Previously published literature has reported ornithine carbamoyltransferase (OTC) deficiency, hematologic malignancy, and the side effects of valproic acid and 5-fluorouracil (5-FU) as the cause of unexplained hyperammonemia in the absence of hepatic dysfunction [6–9]. In this case, the patient was never given any antiepileptic before ICU admission and in ICU stay she received levetiracetam and lacosamide instead of valproic acid to control the epileptic seizures. Hence, this rules out any possible role of valproic acid in raising her serum ammonia levels as supported by the previous evidence [9, 10].

Moreover, the patient had no history of any metabolic disorder so she was not investigated to rule out hyperglutaminemia, or orotic aciduria to diagnose ornithine carbamoyltransferase (OTC) deficiency. However, a previous case supports the possibility of undiagnosed ornithine carbamoyltransferase deficiency resulting in unexplained postpartum hyperammonemia [11]. However, another possible explanation of unexplained postpartum hyperammonemia, in this case, could be enzymatic deficiencies and disorders of the urea cycle that rarely manifest clinically in adulthood until the body goes through physiological stresses or increased catabolism such as during surgery, delivery, or trauma [7, 12, 13]. The postpartum period is a highly catabolic state and unexplained postpartum hyperammonemia is previously reported as a rare outcome among postpartum women with urea cycle enzyme deficiency [13]. Nonhepatic hyperammonemia is now becoming common, with most causes being drug-induced or a genetic inborn error of metabolism. However, it is important to note that genetic testing is not frequently available or done. Further studies and cases are required to support this diagnosis of nonhepatic and non-drug-induced hyperammonemia, especially when genetic predisposition is not known or genetic workup is not available.

Despite these hypotheses, we recommend further research into diagnosing and managing these patients. This case report has blown our minds and forced us to evaluate and study postpartum hyperammonemia. Though cases had been reported in the past, in a third world country such as Pakistan, we should be clinically vigilant about this entity. We have limited diagnostic and therapeutic options in our country, but hyperammonemia needs to be corrected aggressively to avoid death.

Our case had certain limitations. No genetic testing facilities are available at our center. After experiencing an acute deterioration within 3–4 days, we were unable to investigate for an enzymatic deficiency. We did not offer ammonia scavenger therapy to the patient as it is not available in our country. But this case has forced us to think out of the box. Postpartum hyperammonemia is an uncommon illness and requires medical attention for early identification and prompt management.

## Conclusion

Postpartum hyperammonemia is a rare entity. It is a critical illness and underlying metabolic disorders must be evaluated. Early diagnosis and treatment may result in better outcomes and reduced mortality among postpartum women with hyperammonemia.

## Data Availability

Can be reviewed on request after institution approval
